# Anomalous Motion of Charged Domain Walls and Associated Negative Capacitance in Copper–Chlorine Boracite

**DOI:** 10.1002/adma.202008068

**Published:** 2021-03-18

**Authors:** Joseph G. M. Guy, Charlotte Cochard, Pablo Aguado‐Puente, Elisabeth Soergel, Roger W. Whatmore, Michele Conroy, Kalani Moore, Eileen Courtney, Alan Harvey, Ursel Bangert, Amit Kumar, Raymond G. P. McQuaid, J. Marty Gregg

**Affiliations:** ^1^ School of Mathematics and Physics Queen's University Belfast Belfast BT7 1NN UK; ^2^ School of Science and Engineering University of Dundee Nethergate Dundee DD1 4HN UK; ^3^ Institute of Physics University of Bonn Wegelerstrasse 8 Bonn 53115 Germany; ^4^ Department of Materials Imperial College London Exhibition Road London SW7 2AZ UK; ^5^ Department of Physics and Bernal Institute University of Limerick Limerick V94 T9PX Ireland

**Keywords:** boracites, domain walls, negative capacitance

## Abstract

During switching, the microstructure of a ferroelectric normally adapts to align internal dipoles with external electric fields. Favorably oriented dipolar regions (domains) grow at the expense of those in unfavorable orientations and this is manifested in a predictable field‐induced motion of the walls that separate one domain from the next. Here, the discovery that specific charged 90°domain walls in copper–chlorine boracite move in the opposite direction to that expected, increasing the size of the domain in which polarization is anti‐aligned with the applied field, is reported. Polarization–field (*P–E*) hysteresis loops, inferred from optical imaging, show negative gradients and non‐transient negative capacitance, throughout the *P–E* cycle. Switching currents (generated by the relative motion between domain walls and sensing electrodes) confirm this, insofar as their signs are opposite to those expected conventionally. For any given bias, the integrated switching charge due to this anomalous wall motion is directly proportional to time, indicating that the magnitude of the negative capacitance component should be inversely related to frequency. This passes Jonscher's test for the misinterpretation of positive inductance and gives confidence that field‐induced motion of these specific charged domain walls generates a measurable negative capacitance contribution to the overall dielectric response.

## Graphical abstract

During switching, specific charged 90° domain walls in copper–chlorine boracite are seen to move in the opposite direction to that expected, increasing the size of the domain in which polarization is anti‐aligned with the applied electric field. Polarization–field hysteresis loops show negative gradients and measured switching currents confirm the negative‐capacitance contribution to the overall dielectric response.

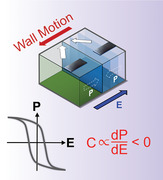

## 1 Introduction

Equilibrium behavior in ferroelectrics can sometimes be surprising. The sequence of phase transformations in Rochelle salt is, perhaps, a good illustration: on cooling, a rather conventional paraelectric‐orthorhombic to ferroelectric‐monoclinic transition initially occurs (at ≈297 K). At lower temperatures (≈255 K^[^ ^1^ ^]^), however, the original high‐symmetry paraelectric‐orthorhombic state is restored. Symmetry associated with this re‐entrant phase transition has unusually, therefore, increased on cooling. Some observations show that this generates a local dip in the heat capacity,^[^ ^1^, ^2^ ^]^ stalling entropy reduction on decreasing temperature.^[^ ^1^ ^]^ Strange symmetry transformations also occur in flux‐grown barium titanate crystals, where highly ordered “Forsbergh Patterns” can first appear and then subsequently disappear, as temperature is monotonically varied.^[^ ^3^, ^4^ ^]^ Most recently, heating has been seen to cause high symmetry labyrinthine ferroelectric domain patterns to give way to lower symmetry stripe arrays: an effect classified as an “inverse transition”.^[^ ^5^ ^]^ Clearly, symmetry changes can therefore occasionally occur in the opposite sense to that normally seen. While fundamental thermodynamic laws are not broken, such cases are unusual, arresting, and worthy of note.^[^ ^6^ ^]^

In contrast, the response of polarization to external electric fields, seen to date in single‐phase proper ferroelectrics, has been thermodynamically unsurprising. During switching, the ferroelectric polarization reorients such that it best‐aligns with the applied field, reducing the free energy of the system by the volume integrated product of the conjugate variables of field (*E*) and the polarization change (Δ*P*).^[^ ^7^ ^]^ Polar realignment is most‐often realized through the nucleation and growth of energetically favored domains (at the expense of unfavored ones) and this is accompanied by a predictable movement of domain walls both individually and collectively.^[^ ^8^, ^9^, ^10^, ^11^ ^]^

Here, we report observations that challenge conventional expectations of dipolar switching. We find both normal and anomalous field‐induced domain wall motion in improper ferroelectric copper–chlorine (Cu–Cl) boracite (Cu_3_B_7_O_13_Cl) single crystals. Charged tail‐to‐tail 90° domain walls move in a conventional sense, increasing the size of domains with polarization best aligned to the applied electric field; charged head‐to‐head 90° domain walls, on the other hand, move to increase the size of domains with strongly anti‐aligned polarization orientations. In effect, when these walls move, the polarization change in the material opposes the electric field creating it, locally increasing the electrostatic component of the free energy in the system and also generating a polarization–field (*P–E*) hysteresis loop that is characterized by a negative gradient at all points. The observed anomalous domain wall motion hence implies an effective negative capacitance throughout the switching cycle. Such a negative capacitance has been explicitly demonstrated, by combining observations of domain wall motion, under bias, with measurements of the switching charge developed as a result, using charge gradient microscopy (CGM).

## 2 Domain Structure and Polar Orientations in Boracite Single Crystals

Accurately establishing the nature of the domain states (and associated dipole orientations), in the specific Cu–Cl boracite crystals investigated herein, is critical for making valid statements about electric field‐induced changes in polarization. We have used three different sources of information to fully characterize them: i) shear strains apparent through surface topographic mapping, ii) domain wall conduction measurements, and iii) piezoresponse force microscopy (PFM).

Cu–Cl boracite (Cu_3_B_7_O_13_Cl) undergoes an improper ferroelastic (and accompanying improper ferroelectric) phase transition from the cubic space group $F\bar{4}3c$ to orthorhombic *Pca*2_1_ at around 363 K.^[^ ^12^ ^]^ The transition involves a modest uniaxial shear strain, changing the geometry of an opposing pair of {100}_cubic (c)_ faces from a square into a rhombus, while leaving the other faces of the unit cell unchanged.^[^ ^13^ ^]^ Associated atomic displacements result in the development of a spontaneous polarization (P_s_) along <100>_pseudocubic (pc)_ directions (perpendicular to the sheared faces of the unit cell) with a magnitude estimated to be ≈1.85 μC cm^−2^.^[^ ^14^ ^]^ There are six equivalent ways in which the shear strain can develop, with each generating one specific direction in polarization; hence, only six possible domain states can form as a result of the symmetry breaking.^[^ ^15^, ^16^, ^17^ ^]^

**Figure 1:**
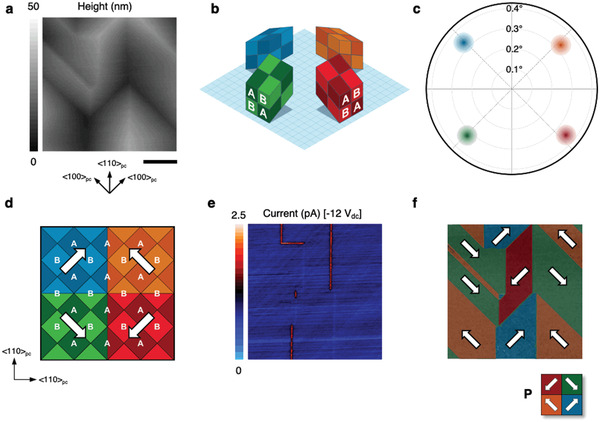
a) Local topography of the Cu–Cl boracite crystal surface as measured using atomic force microscopy. The relevant crystallographic axes are given and the scale bar measures 5 µm. b) Four (of six) color‐coded unit cell orientations associated with the low‐temperature ferroelectric/ferroelastic phase, where an “A/B” sublattice is identified within each variant. The shear angle is exaggerated by a factor of ≈100. c) Pole plots of the vector normals to the crystallographic planes, associated with the surface corrugations observed on the boracite crystals, at room temperature. There are only four distinct poles (associated with four shear variants), ≈90° apart, with consistent ≈0.3° inclination angles away from the mean surface normal. The color‐coded halos represent the measurement uncertainty. d) Chemical‐structural compatibility of elastic domains enforces a discontinuity in polarization across {110}_pc_‐type interfaces. e) Spatially resolved current map from the region mapped in (a); –12 V_dc_ was applied to the bottom electrode. Domain boundaries with enhanced and reduced conductivity with respect to the bulk are those oriented along the {110}_pc_ planes. f) Corrected lateral mode piezoresponse for the same region. The legend represents the four in‐plane polarized domain states with their orientations labelled by white arrows. Information from both (e) and (f) indicates that the head‐to‐head charged domain walls are electrically insulating, while the tail‐to‐tail ones are conducting.

The single crystal boracite plates, examined in this research, were oriented with polished top and bottom faces parallel to {100}_pc_; transmission electron microscopy confirmed this orientation and showed that the internal crystallographic structure of the Cu–Cl boracite was consistent both with other members of the boracite family (for which atomic coordinates within the unit cell are explicitly known) and with the equilibrium room temperature atomic distribution determined using density functional theory (Figure S1, Supporting Information). On heating and cooling through the ferroelastic‐ferroelectric phase transition, distinct topographic corrugations were seen to develop on the surface of the bulk crystal (imaged using atomic force microscopy (AFM), **Figure** **1**a). The inclined nature of these corrugations indicated that only the four ferroelastic domain variants with polarizations parallel to the crystal surface had formed (Figure 1b). Vector normals from surface planes (plotted in Figure 1c) showed all to be inclined at approximately the same angle away from what had been the polished surface normal (suggesting ≈0.3° for the angle associated with the spontaneous shear); this is entirely consistent with the formation of only the four shear variants considered schematically in Figure 1b. Shear variants were found to abut along <100>_pc_ and <110>_pc_ line vectors on the crystal surface (Figure 1a), consistent with the expected {100}_pc_ 180° and {110}_pc_ 90° domain walls, respectively. These domain wall planes are oriented perpendicularly to the crystal surface, and this is explicitly confirmed by through‐focus optical microscopy (see Movie S1 and Figure S2, Supporting Information). The {110}_pc_ 90° domain walls in the boracite system are unique, as the combination of elastic compatibility and chemical‐structural continuity demands either head‐to‐head or tail‐to‐tail polar discontinuities (Figure 1d).^[^ ^13^, ^18^ ^]^ This requirement is explicitly demonstrated by group theory analysis (see table 27 in ref. ^[^ ^17^ ^]^). Uncharged (head‐to‐tail) {110}_pc_ 90° domain walls are hence symmetry‐forbidden.

Domain walls, which support polar discontinuities, often generate electrical transport behavior that is distinct from the bulk^[^ ^19^, ^20^, ^21^, ^22^, ^23^ ^]^ and that was also found to be the case in the Cu–Cl boracite crystals examined:^[^ ^24^ ^]^ Figure 1e, shows clearly that the 90° {110}_pc_ domain boundaries are associated with dc conductivity anomalies. PFM (Figure 1f) indicates that tail‐to‐tail 90° domain walls are responsible for enhancement and head‐to‐head walls for suppression of conductivity. The lack of out‐of‐plane PFM amplitude is consistent with the entirely in‐plane polarization inferred from all of the observations made above. It should be noted that, as found in previous work,^[^ ^24^ ^]^ analysis of the PFM contrast in the boracites is more complex than that in more conventional ferroelectrics; the methodology used for interpreting this PFM data is discussed in Figures S3,S4 and associated Note S1, Supporting Information.

## 3 Field‐Induced Motion of the 90° Domain Walls

**Figure 2:**
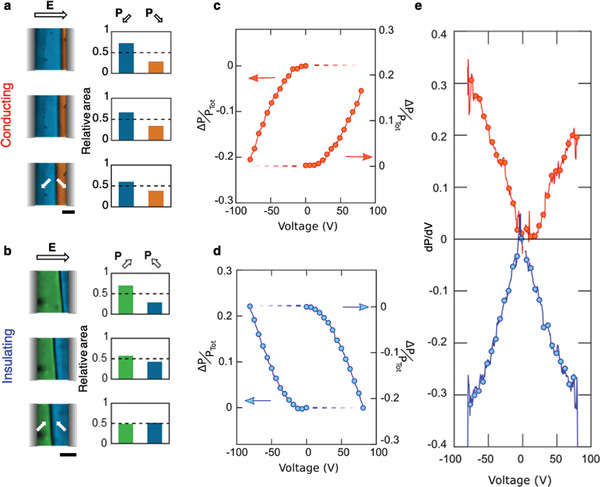
a,b) False‐colored optical microscopy images demonstrating the motion of conducting tail‐to‐tail (a) and insulating head‐to‐head 90° (b) domain walls under a switching bias (the field direction is indicated by the black‐outline white arrow). The accompanying volumetric change in domain population within the interelectrode gap is plotted to the right of each microscopy image. The domain orientations are represented by the white arrows. The scale bar measures 35 µm. c,d) Tracking the change in polarization, Δ*P*, allows for plotting of a normalized positional hysteresis for conducting (c) and insulating walls (d). e) The derivative of the change in polarization with respect to the applied voltage for both types of charged wall. The derivative is proportional to the contribution from domain wall motion to the dielectric permittivity of the system. It is clear that anomalous wall motion, leading to the growth of the domain anti‐aligned with the electric field, is associated with a negative derivative at all levels of applied voltage, implying a consistently negative capacitance contribution.

As in previous work,^[^ ^24^ ^]^ we used the application of point‐pressure (of order 1 GPa) to write domain patterns with extended {110}_pc_ 90° domain wall sections; thin film strip electrodes (gold) were deposited such that individual pressure‐written walls lay within a ≈200 µm interelectrode gap and were oriented with their surface traces approximately parallel to the electrode–dielectric edges (**Figure** **2**a,b). In most cases, only two domain states, separated by one domain wall, were contained within the interelectrode gap. The motion of the domain walls (and the associated growth and contraction of domains) was then monitored, under an optical microscope, as sweeps in potential difference were applied. In general, all such switching experiments were performed at slightly elevated temperatures (≈360 K), to enhance domain wall mobilities. It is important to note that optical imaging was performed in transmission mode and hence that the projection of the entire domain wall through the thickness of the crystal was captured. The observations described below are hence not purely surface‐related, but genuinely reflect volume changes in domains through the entire crystal thickness.

The electric‐field‐induced motion of 90° tail‐to‐tail and head‐to‐head domain walls were found to be surprisingly similar, insofar as both move in the same direction (against that of the applied field—see Movies S2 and S3, Supporting Information) and for both, the domain wall speeds were found to be constant (at constant applied switching field). In one instance, pressure writing created a pair of parallel 90° domain walls that were closely separated and could both therefore be simultaneously captured between the electrodes, with one domain wall being of conducting tail‐to‐tail type and the other being of the insulating head‐to‐head type. Consistent with observations made on individual walls, applied electric fields caused both to move in the same sense (see Movie S4, Supporting Information). We note that the domain walls were only seen to move whenever the electrode bias was turned on and their movement reversed when the sense of bias reversed. Hence spurious external stresses from mechanical clamping or probe contact with the thin film electrodes can be discounted as the primary driving force for the domain wall motion seen.

For the conducting tail‐to‐tail walls, the development of polarization with field is entirely conventional and thermodynamically expected (Figure 2a): the domain variant with a polarization component parallel to the applied field expands at the expense of the variant with an antiparallel component and hence the net polarization change (Δ*P*) is in phase with the field (*E*); an electrostatic contribution of the form −|*E*||Δ*P*| is therefore made to the free energy of the system and this is characteristic of normal switching behaviour.

The field‐induced movement of the insulating head‐to‐head wall, on the other hand, cannot be so easily explained. In this case, growth of the domain with a polarization component anti‐aligned to the field occurs and the domain with a field‐aligned component contracts. In other words, the development of polarization is out‐of‐phase with the applied field: positive field results in negative polarization development and vice versa. Thermodynamically, this means a free energy contribution (increase) of +|*E*||Δ*P*| due to the domain changes, and an overall electrostatic energy cost of approximately +2|*E*||Δ*P*| over that which would have resulted from conventional behaviour (if the head‐to‐head wall had moved the other way). Finite element simulations (in which all the forms of the electrodes used in various incarnations of the experiments in which anomalous motion was mapped) allowed for a more rigorous evaluation of the electrostatic work done during switching (Figure S5, Supporting Information). In all cases, it was found to be positive for the head‐to‐head walls.

Figure 2b summarizes results from mapping the electric‐field‐induced anomalous motion of a 90° head‐to‐head wall. It should be reiterated that the crystallographic planes, along which these domain walls lie, are perpendicular to the surface (Figure S2, Supporting Information) and that imaging was done in transmission mode; hence the change in position of the line trace of the domain wall can be taken as a proxy for the change in volume of the domains within the interelectrode gap, induced by the applied field. By integrating the surface area of the domains between the electrodes, the sum of the individual polarization components parallel and anti‐parallel to the applied electric field can therefore be determined (Figure 2a,b). The difference in the areal populations of these domains reflects the overall polarization in the co‐planar capacitor structure and so the information can be used to construct effective *P–E* hysteresis plots (Figure 2c,d). The *P–E* loop shown in Figure 2d is associated with the anomalous motion of the wall, and has an inverted shape compared to the classical hysteresis loop obtained for the conducting tail‐to‐tail wall (Figure 2c).

We note, parenthetically, that superficially similar “positional” hysteresis loops have been published, showing the motion of 90° ferroelastic domain walls in BaTiO_3_, by Fousek and Brezina (Figure 10b in ref. ^[^ ^25^ ^]^). Importantly, however, mirror reflections of these loops were certainly not due to anomalous domain wall motion; instead, progressive 180° reversal of the polar orientations of all domains caused field‐induced 90° wall motion to reverse. Hence, while wall motion changed, the sense in which field‐aligned polarization developed during switching was conventional at all points.

## 4 Negative Capacitance and Measurements using Charge Gradient Microscopy

The *P–E* hysteresis, associated with the anomalous 90° domain wall motion in the Cu–Cl boracite (Figure 2d), indicates that the gradient of the polarization with respect to field (d*P*/d*E*) is negative throughout the range of fields applied (Figure 2e). A non‐transient negative capacitance is therefore implied, that should be manifest in directly measurable functional properties: by mapping the switching currents associated with field‐driven domain wall motion, for example. Initial attempts to make such measurements were frustrated by relatively low field‐driven domain wall propagation speeds, combined with the inherently modest spontaneous polarization in the material itself. Even at the maximum bias levels we could apply, before the domain walls “broke‐up” or significant leakage currents developed (≈100 V), domain wall speeds were only found to be of the order of 5 µm s^−1^. We were unable to clearly resolve the resulting modest switching currents, as the rate of polarization reversal was just too low.

A much greater rate of change in polarization, produced by a much more rapid relative motion between domain walls and sensing electrodes, was therefore needed; this was made possible by changing the reference frame for the experiment: instead of observing switching currents in the rest frame of the electrodes, we measured them in the rest frame of the domain wall; in other words, relative motion between the electrodes and walls was realized by moving the electrodes, rather than the walls, using CGM.

CGM is a scanning probe microscopy technique (first reported by Hong et al. in 2014^[^ ^26^ ^]^), which allows spatial gradients in ferroelectric bound charge to be directly imaged, by monitoring currents passing through an earthed passive conducting AFM tip (usually solid platinum), pressed strongly into contact and rapidly scanned across the sample surface. Whether the technique involves changes in the screening charges at the surface of the metallic tip, or the “collection” of screening charge already aggregated on the ferroelectric free surface, is still a matter of debate^[^ ^26^, ^27^, ^28^, ^29^ ^]^ (see Note S2, Supporting Information). In any case though, the integration of the measured currents across stationary domain walls in CGM has been shown to reflect the switching charge associated with polar reorientation under the moving tip.^[^ ^26^ ^]^ We have independently confirmed Hong et al.'s observations and have been able to link CGM current signals, resulting from tip motion over static domain walls, to those expected from an equivalent domain wall motion relative to fixed electrodes (changing the reference frame for the experiment), as illustrated in Figures S6,S7, Supporting Information. In z‐cut periodically poled LiNbO_3_ single crystals, therefore, CGM currents (generated with a moving tip and fixed domain walls) match the form of those predicted from finite element modeling, when a wall moves at constant velocity toward, under and away‐from a pair of static short‐circuited electrodes.

**Figure 3:**
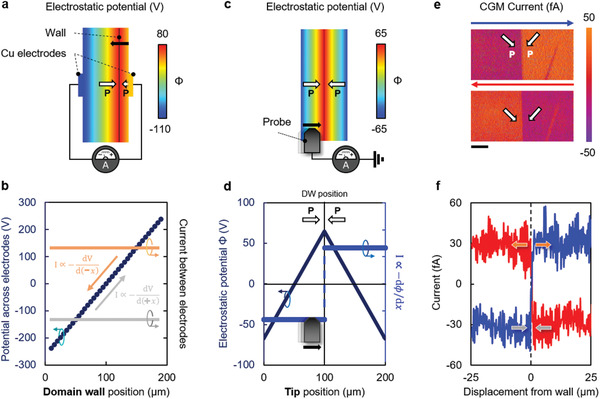
a) Finite element modeling of the local electrostatic potential landscape near a moving 180° head–head domain wall in PZT (importantly, no external bias is being applied in the model—the wall motion is not being field‐driven). b) Potential difference between the Cu electrodes varies linearly as a function of domain wall position; the associated external current is constant (and sign dependent) for a constant domain wall velocity. c) The same relative movement between the electrode and the wall is reproduced in charge gradient microscopy (CGM) by moving the tip in the opposite sense with respect to the static domain wall. d) Electrostatic potential sensed by the tip when traversing across the domain wall, along with the associated current. This current (which is proportional to the spatial derivative of the potential) is constant and negative for a constantly reducing tip–wall separation, and positive for a constantly increasing tip‐wall separation; both of these current signals are fully expected if the tip moves at constant speed over the domain wall. e) Spatially resolved CGM current data for a 90° charged insulating head–head domain wall in Cu–Cl boracite. The blue and red arrows indicate the probe scanning direction (trace and retrace, respectively). f) Averaged CGM current line profiles corresponding to trace (blue) and retrace (red). The arrows illustrate that when the relative separation between probe (electrode) and wall constantly increases, current is positive, whereas for a relative separation that is constantly decreasing, the current is negative.

CGM has not yet been explicitly reported for domain patterns with all‐in‐plane polarization, despite the fact that meaningful contrast should be expected. To illustrate, **Figure** **3**a presents insight from further 2D finite element simulations, in which a charged 180° domain wall lies within an interelectrode gap and is oriented parallel to the electrode–ferroelectric interface. As the domain wall moves, the potential difference between the electrodes changes linearly as a function of wall position (Figure 3b). Hence, if the wall moves at constant speed (as found in our switching experiments), the associated external current, driven by this changing potential, should also be constant (Figure 3b). Importantly, the sign of this current depends on whether the wall is moving toward, or away from, the sensing electrode. As a consequence, on changing perspective to recreate the same relative motion between electrode and domain wall, but now within the rest frame of the domain wall (in an equivalent CGM geometry), one also expects a constant current as the tip moves. Importantly, the sign of this current should flip as the tip passes over the domain wall and the relative motion between tip and wall changes from them approaching one another, to them moving apart (as shown in Figure 3c,d). This current response is exactly that observed experimentally: Figure 3e,f shows CGM maps and current signals across the boracite head‐to‐head 90° wall. The current of constant magnitude which flips sign across the wall is self‐evident. This appears to be a generic finding, confirmed by taking images and current profiles across sections of head‐to‐head 180° charged domain walls in a LiNbO_3_ single crystal, with polarization in‐plane (Figure S8, Supporting Information).

**Figure 4:**
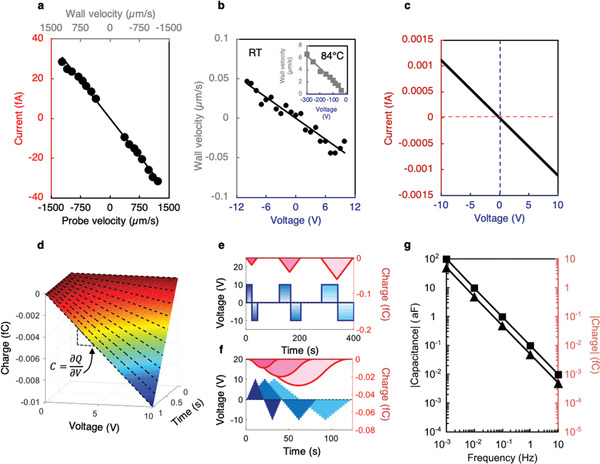
a) The CGM current under different scan‐rates, measured either when moving toward (positive velocity) or away from (negative velocity) the 90° in‐plane head‐to‐head domain wall in the Cu–Cl boracite. b) The velocity associated with the anomalous field‐driven motion of these head‐to‐head walls, as a function of switching bias applied to a positive surface electrode (the main panel data have been determined from in situ scanning probe microscopy at room temperature, while the inset shows data taken from in situ optical microscopy, performed at 84 °C, using geometries similar to those associated with Figure 2 and the Movies S2–S4, Supporting Information). c) By combining data from (a) and (b), the tip current (at a given velocity) can be expressed as a function of the switching voltage needed to induce the same relative motion between tip and domain wall. d) Given that the wall velocities are constant in time (for each driving voltage), the current–voltage relation given in (c) can be unpacked into a charge–time–voltage plot, allowing the capacitance to be explicitly determined, as a function of the time over which relative motion between electrode and wall occurs. e) Since the wall displacement is a linear function in time (the wall velocity is constant for each applied switching bias), the total charge collected at a sensing electrode can be calculated for different square pulse frequencies and hence modeled for more complex ac signals (such as the triangular pulses in (f)). g) The accumulated charge and hence negative capacitance can then be expressed as a function of ac signal frequency (squares indicate capacitance and charge from square waveforms and triangles from triangular waveforms). Note the gradient in the log–log plot shows that the magnitude of the negative capacitance is inversely related to frequency, passing Jonscher's test for negative capacitance being inappropriately attributed to inductance.

We should now try to understand the implications of these CGM measurements for negative capacitance. From Figure 2, we see that, for a notionally “positive” applied electric field, the head‐to‐head domain wall moves in a “negative” direction. This movement is against the field, and reduces the distance between wall and positively biased electrode through which the switching current develops. In the rest frame of the domain wall (Figure 3c), the same event (same relative movement between wall and electrode) is reproduced when the conducting CGM tip moves toward the wall (the electrode wall separation is reduced). Hence, the CGM current measured, when the tip moves toward the head‐to‐head wall, is the same sign as that of the switching current, in a conventional fixed‐electrode field‐driven switching experiment. Crucially, this current is measured to be negative (Figure 3e,f); moreover, it is the same, irrespective of whether the tip approaches the wall during the trace or the retrace scan. Its magnitude can be measured explicitly, as a function of the tip velocity (**Figure** **4**a). Equally, and importantly, the wall velocity as a function of applied voltage, determined through both optical and PFM imaging during field‐induced switching experiments (Figure 4b), is already known. Armed with these two pieces of information, the form of the switching current obtained through CGM can be meaningfully mapped onto the applied bias that produces the same relative motion between domain walls and sensing electrodes (during a conventional switching experiment). In this way, the relationship between applied voltage and switching current, resulting from anomalous domain wall motion, can be deduced (Figure 4c).

As noted previously, for each value of the applied field, the domain wall velocity during switching was found to be constant; hence, the switching current versus applied voltage information presented in Figure 4c can be unpacked to create a charge–time–voltage plot (Figure 4d). One can see from this that, if different voltages are applied for the same duration, the resulting charge–voltage relationship can be extracted. Such plots (keeping the time over which the voltage is applied to be constant) are linear in all cases and have negative gradients (see the charge–voltage dotted line functions in Figure 4d). Since this gradient is, by definition, the capacitance associated with the anomalous domain wall motion, its negative sign has therefore been experimentally demonstrated. The time‐dependence of the charge build‐up on the sensing electrodes, due to the uniform domain wall movement under constant bias, also allows us to infer the frequency dependence of this negative capacitance component, under a notional applied ac driving voltage (Figure 4e): its magnitude is inversely proportional to frequency (Figure 4f). This behavior passes Jonscher's test for the inappropriate assignation of a measured positive inductance as being due to a negative capacitance.^[^ ^30^ ^]^

## 5 Negative Capacitance and the Possibility of Local Field‐Reversal

Behaviors coherent with negative capacitance have been observed in a variety of systems, such as semiconductor devices (e.g., p–n junctions or Schottky diodes), quantum well infrared detectors,^[^ ^31^ ^]^ and solid electrolytes.^[^ ^30^ ^]^ In these systems, the apparent negative capacitance is related to retardation of the applied field. In ferroelectrics, the same retardation has been noted in some reports.^[^ ^32^ ^]^ Although these systems present a macroscopic behavior that can be described by negative capacitance, they do not show intrinsic negative capacitance. From an energetic point of view, negative capacitance requires an effective negative curvature of the free energy. In ferroelectrics, this has been achieved in static and transient (switching) regimes. In both cases, a local electric field opposing the macroscopically applied one develops,^[^ ^33^ ^]^ due to the presence of a static capacitance^[^ ^34^, ^35^ ^]^ or during switching under the application of large electric fields.^[^ ^34^, ^36^, ^37^, ^38^ ^]^ In all cases, it is worth noting that, macroscopically, the overall capacitance of the system remains positive.^[^ ^33^ ^]^

**Figure 5:**
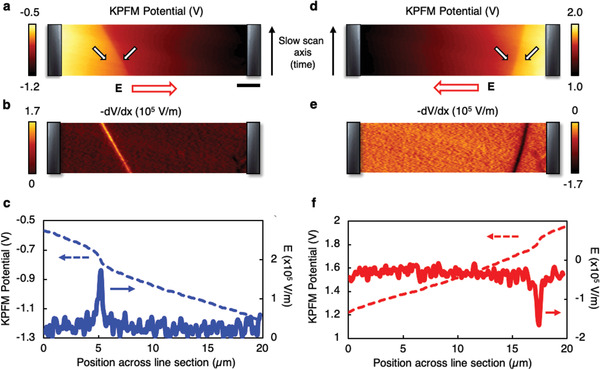
a) Kelvin probe force microscopy (KPFM) of an insulating domain wall, when subject to an applied bias from co‐planar electrodes (marked by the gray motifs straddling each site of the panel). The field direction is indicated by the bold red arrow, and polarization within each domain is indicated using the black‐outlined white arrows. The apparent inclination of the wall is due to the finite time taken to complete the KPFM scan, where the slow‐scan direction is indicated by the thin black arrow on the right‐hand side of the panel (again indicating that the wall moves against the field). The scale bar measures 2.5 µm. b) The negative of the derivative of (a) is an effective map of the local E‐field profile, revealing a field‐peak that correlates spatially with the domain wall. c) Representative line profiles are extracted from (a) and (b), which clearly show a monotonic decrease in the KPFM potential (moving left to right). d) The same wall is now subject to a bias of reverse polarity, where the field direction is again indicated by the red‐outline arrow. e) The negative derivate (field map) reveals a similar anomaly that correlates with the position of the wall throughout the scan. f) Associated line profiles for (d) and (e) reveal a monotonic increase in the KPFM potential. The monotonic nature of (c) and (f) precludes the notion of local field reversal at the domain wall.

To check the local field behavior in the boracite during anomalous domain wall motion, in situ Kelvin probe force microscopy (KPFM) imaging was performed across a 90° head‐to‐head wall, as it moved between fixed electrodes, under an applied bias (**Figure** **5**a,b,d,e). As the slow scan axis was not disabled, the apparent inclination of the domain walls is a result of bias‐induced movement during imaging. KPFM measures the surface potential, from which the local electric field can be directly determined, by extracting the negative of the potential gradient at each point (Figure 5c,f). As can be seen, while the local electric field determined in this way varies in magnitude, it maintains a uniform direction in both the domains and across the domain wall (the same as that of the applied field). In contrast to previous work,^[^ ^33^ ^]^ the negative capacitance contribution from the anomalous domain wall motion in Cu–Cl boracite cannot therefore be ascribed to a local electric field reversal.

## 6 Thermodynamics of Anomalous Domain Wall Motion

We have not been able to explicitly rationalize the thermodynamic reasons for the observed anomalous domain wall motion and its associated negative capacitance. In principle, one may start developing an understanding by considering Landau–Ginzburg–Devonshire–Levanyuk free energy expansions for improper ferroelectrics, which are typically (given in scalar form) as follows:^[^ ^39^, ^40^, ^41^ ^]^

(1) $$\begin{array}{c} G=G_{0}+\underset{n}{\sum }a_{n}q^{2n}+\underset{m}{\sum }A_{m}P^{2m}+\underset{i,j}{\sum }\alpha_{ij}q^{i}P^{j}-E\cdot P+\text{other}\;\text{conjugate}\;\text{variable}\;\text{pairs} \end{array}$$

where *q*  is the primary order parameter, *P* is the polarization, and *E* is the electric field; *a_i_*, *A_m_* and α_*ij*_ are prefactors where *a*_1_ is the temperature‐variable prefactor containing the (*T* − *T*_C_) term, *T* is temperature and *T*_C_ is the critical temperature associated with the phase transition. For the specific case of boracites, no consensus on the exact form of this free energy expansion has yet been reached^[^ ^42^, ^43^, ^44^, ^45^, ^46^, ^47^, ^48^ ^]^ and hence the values of the different prefactors needed are completely unknown. Without any constraints, conditions for the prefactors α_*ij*_ can be found such that it is thermodynamically favourable for the head‐to‐head wall to move “anomalously”. However, the free energy expansion, as expressed in Equation (1), in terms of structural parameters, is insensitive to the spatial distribution of domains. Thus, expressions that facilitate anomalous motion of head‐to‐head walls, through stabilization of polar regions anti‐aligned to the applied field, must automatically demand similarly anomalous motion of tail‐to‐tail walls (as anti‐aligned domains must be uniformly energetically favored) and this is obviously not commensurate with our observations. However, accounting for the distribution of free carriers would break the symmetry between head‐to‐head and tail‐to‐tail domain walls. The associated energy term would be inversely proportional to the local density of states, which would be different for positive and negative carriers.^[^ ^49^ ^]^ It is therefore conceivable that a complete expansion including both structural and electronic degrees of freedom might be able to accommodate the observed behavior.

Recently, Luk'yanchuk et al.^[^ ^50^ ^]^ showed theoretically that anomalous domain wall movement could occur in circular nanocapacitors where an increase in electrostatic energy could be offset by a reduction in domain wall area (and hence total domain wall energy). While in the boracites the domain wall area of the field‐driven wall is not obviously reduced, a similar possible energy offsetting mechanism might be facilitated by long‐range domain reorganization. Indeed, long‐range microstructural changes were reversible and repeatably correlated with the direction of the applied field, suggesting that they were fully coupled to the local charged domain wall motion (Movie S5, Supporting Information). Therefore, the possibility that a localized increase in the electrostatic free energy (within the interelectrode gap) may be offset by energy reductions elsewhere in the microstructure should be taken seriously. However, this notion has not been fully developed yet and our initial attempts suggest that it is non‐trivial.

## 7 Summary and Outlook

While the energetics responsible for anomalous domain wall motion in the Cu–Cl boracite system are somewhat uncertain, the ability to induce polarization that is anti‐aligned with the electric field creating it is an unprecedented observation; the fact that this generates a measurable negative capacitance contribution to the overall dielectric response should be of great interest fundamentally and for device applications, in which negative capacitance can be exploited.

## 8 Experimental Section

### Sample Preparation and Atomistic Structure Models

The single crystals of Cu_3_B_7_O_13_Cl used in this study, several millimetres in size, were grown by the sealed‐ampoule vapor phase growth technique described elsewhere.^[^ ^51^ ^]^ These were X‐ray oriented, sliced into 0.5 mm‐thick {100}_pc_ oriented plates using a diamond saw and polished using successively finer grades of diamond paste, finishing with colloidal silica (Syton). The VESTA software package^[^ ^52^ ^]^ was used to make the atomistic structure models shown in Figure S1, Supporting Information.

### Scanning Probe Microscopy Characterization of Boracite Crystals

Scanning probe microscopy studies of the boracite crystals were carried out using two separate systems: a Veeco Dimension 3100 AFM system (equipped with Nanoscope IIIa controller) and an Asylum Research MFP‐3D Infinity AFM system. For PFM measurements, the Veeco system was used in conjunction with an EG&G 7265 lock‐in amplifier, whereas the Asylum system used a proprietary internal lock‐in amplifier. Commercially obtained Pt/Ir‐coated Si probes (Nanosensors model PPP‐EFM) were used for all measurements. For PFM measurements made far from the tip‐contact mechanical resonance, a 5 V, 20 kHz a.c. probing signal was used. Near‐tip‐contact resonance measurements were made at probing frequencies of ≈330 kHz and ≈660 kHz for vertical and lateral PFM modes, respectively. Spatially resolved current mapping was carried out at room temperature using the Veeco system equipped with a Bruker Tunnelling AFM (TUNA) module. Currents were monitored while voltages up to −12 V_dc_ were applied to the base of the crystal with the tip grounded. KPFM was used to measure the local surface potential across charged domain walls in Cu_3_B_7_O_13_Cl and was performed using the Asylum system. A two‐pass technique, KPFM involved rastering a probe across the sample surface where, on the first pass, the topography was mapped using conventional tapping (or AC) mode before withdrawing to a fixed height (50 nm) above the surface in the second pass. During the second pass specifically, a d.c. bias was applied to the probe such that any changes in the bias, due to the influence of local electrostatic forces, were monitored and plotted as a 2D, spatially resolved surface potential map. CGM allowed to spatially map local variations in electrical charge on the nanoscale, specifically across domain walls in a commercially available sample of periodically poled lithium niobate (LNO), an in‐plane polarized sample of LNO, and Cu_3_B_7_O_13_Cl. In the context of this work, CGM was used to map the displacement current associated with unscreened polarization by quickly rastering a grounded, conducting AFM probe across the grounded sample with a large deflection setpoint (i.e., pressure) such that screening charges were scraped away, resulting in the formation of mirror charges on the probe as it attempts to maintain electrical ground when scanning across the now unscreened sample surface.

In order to map the vector normals of the crystal surface planes onto a stereographic projection plot, a background plane fit was first subtracted from the raw AFM measured topographical map (flattening). For each topographically distinct surface, the coordinates of all points were fitted to a plane and the vector normal was calculated.

### Electric Field‐driven Motion of Charged Domain Walls

Charged conducting/insulating domain walls were site‐specifically created at elevated temperatures in Cu_3_B_7_O_13_Cl crystals using a bespoke spring‐loaded pressure rig^[^ ^24]^. Approximately 40 nm‐thick Au patterned electrodes were then sputter‐deposited on either side of a stress‐written charged domain wall so that planar electric fields could be applied. The crystal was then suspended across a hole in the center of a ceramic heating element to allow the temperature of the crystal to be controlled (via a platinum temperature sensor connected to a Thorlabs Model TC200 Controller) while polarized light microscopy was carried out in transmitted light. Long‐period triangular voltage waveforms were applied to the electrodes with peak voltages as large as ±100 V, using a Keithley Model 237 source ‐ measure unit, and the resulting charged domain wall motion between the co‐planar electrodes was recorded optically. Hysteresis loops were reconstructed by estimating the net polarization change associated with the relative change in surface area of the two domain variants observed optically within the electrode gap as a function of applied electric field. For the positive branch of the hysteresis loop, polarization change was measured relative to the initial domain configuration before the field was applied. For the negative branch, the polarization change was measured relative to the domain configuration observed at the point where the field polarity was reversed.

### Scanning Transmission Electron Microscopy

An electron‐transparent cross‐section of the materials was prepared for STEM using a dual‐beam focused ion beam integrated scanning electron microscope (Thermo‐Fisher Scientific FEI Helios G4 CX model). The specimen was mounted onto an Omniprobe copper‐based lift‐out grid. The thinning of the sample was done in four steps as detailed in ref. ^[^ ^53^ ^]^. The STEM analysis was performed using a Thermo‐Fisher Scientific double tilt TEM holder in the Thermo‐Fisher Scientific FEI double aberration‐corrected monochromated Titan Themis Z at the University of Limerick. The microscope was operated at 300 kV. The imaging mode was STEM annular bright field using the DF2 camera at camera length 115 mm and a 50 µm C2 aperture.

## Conflict of Interest

The authors declare no conflict of interest.

## Supporting information

Supporting Information

Supporting Information

Supporting Information

Supporting Information

Supporting Information

Supporting Information

Supporting Information

Supporting Information

## Data Availability

The data that support the findings of this study are available from the corresponding author upon reasonable request.
