# Supplementary material for: Anomalous Motion of Charged Domain Walls and Associated Negative Capacitance in Copper–Chlorine Boracite
Source: Adv Mater. 2021 Mar 18;33(16):2008068. doi: 10.1002/adma.202008068 (PMC11469175; doi:10.1002/adma.202008068)
Supplement: Supplementary file 1 — Supporting Information [file ADMA-33-2008068-s001.pdf]

© 2021 Wiley-VCH GmbH

ADVANCED MATERIALS

Supporting Information

for *Adv. Mater.,* DOI: 10.1002/adma.202008068

Anomalous Motion of Charged Domain Walls and Associated Negative Capacitance in Copper–Chlorine Boracite

*Joseph G. M. Guy, Charlotte Cochard, Pablo Aguado-Puente, Elisabeth Soergel, Roger W. Whatmore, Michele Conroy, Kalani Moore, Eileen Courtney, Alan Harvey, Ursel Bangert, Amit Kumar, Raymond G. P. McQuaid, and J. Marty Gregg**

Supplementary Information

Anomalous Motion of Charged Domain Walls and Associated Negative Capacitance in Copper-Chlorine Boracite

Joseph G. M. Guy[1], Charlotte Cochard[1], Pablo Aguado-Puente[1], Elisabeth Soergel[2], Roger W. Whatmore[3], Michele Conroy[4], Kalani Moore[4], Eileen Courtney[4], Alan Harvey[4], Ursel Bangert[4], Amit Kumar[1], Raymond G. P. McQuaid[1] and J. Marty Gregg[1*]

[1] School of Mathematics and Physics, Queen's University Belfast, Belfast, BT7 1NN, U.K.

[2] Institute of Physics, University of Bonn, Wegelerstrasse 8, 53115 Bonn, Germany

[3] Department of Materials, Imperial College London, Exhibition Road, London SW7 2AZ, U.K.

[4] Department of Physics & Bernal Institute, University of Limerick, Limerick, Ireland

*email: m.gregg@qub.ac.uk

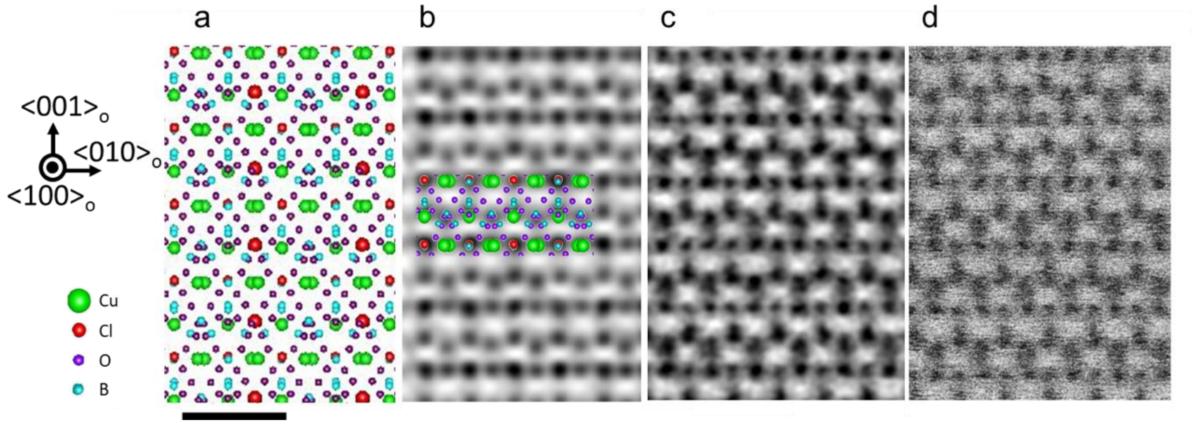

Supplementary Figure S1| Structure of Cu-Cl boracite (a) Atomic structure of boracite (first principles simulations – see comment below) along with (b) the simulated annular bright field (ABF) image expected under the imaging conditions used to obtain (c) Fourier filtered and (d) raw images of Cu-Cl boracite. The scale bar (bottom left) represents 1 nm. First-principles simulations were carried out within the spin polarized density functional theory as implemented in the Quantum-Espresso suite [Giannozzi *et al.*, QUANTUM ESPRESSO: a modular and open-source software project for quantum simulations of materials, *J. Phys.: Condens. Matter* **21** 395502 (2009)]. The revised Perdew-Burke-Ernzerhof generalized gradient approximation [Perdew *et al.*, Restoring the Density-Gradient Expansion for Exchange in Solids and Sufaces, Phys. Rev. Lett. 100, 136406 (2008)] was used for the exchange-correlation functional and a Hubbard-like on-site repulsion of U=4 eV was introduced for the Cu ions. Atomic structure was relaxed starting from the coordinates reported for Mg-Cl structure [Ito *et al.* The crystal structure of boracite. *Acta Cryst*. **4**, 310-316 (1951)] with random displacements $\delta r < 0.09$ Å, until the forces were smaller than 0.02 eV/ Å. The simulated STEM images used the atomic position coordinates given in F. R. Thornley *et al.* Structural studies of Cu-Ci-boracite, *Ferroelectrics*, **13**, 357-359 (1976); these coordinates turned out to be almost identical to those resulting from the first-principles modelling done. STEM simulations used "Dr Probe" (J. Barthel, Dr. Probe: A software for high-resolution STEM image simulation, *Ultramicroscopy*, **193**, 1-11 (2018)).

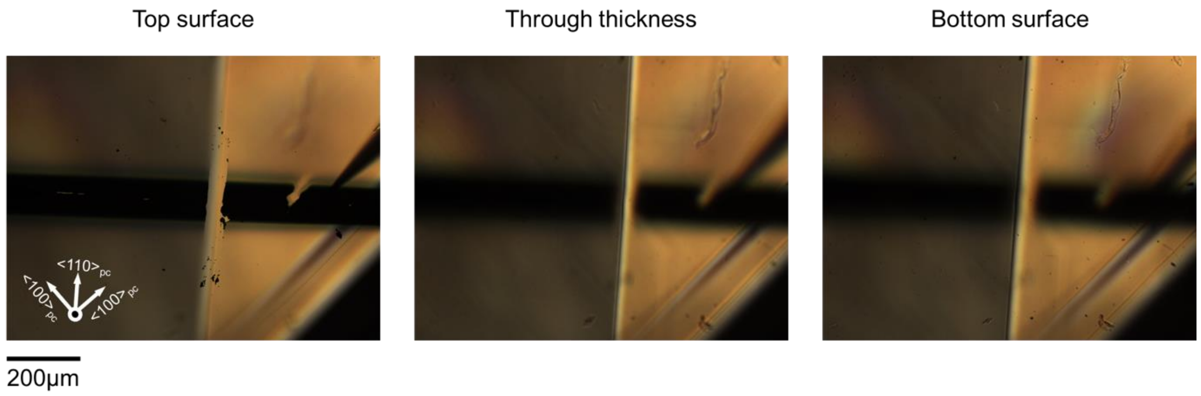

Supplementary Figure S2| Domain walls through the thickness. Transmission polarized light microscopy of a head-to-head 90° charged insulating wall focused at three positions (top and bottom surface and approximately half-way through the crystal). The position of the wall with respect to the top surface electrodes (which appear as two black strips in the above optical micrographs) remains the same as the focus is adjusted, confirming that the domain wall is orthogonal to the sample surface.

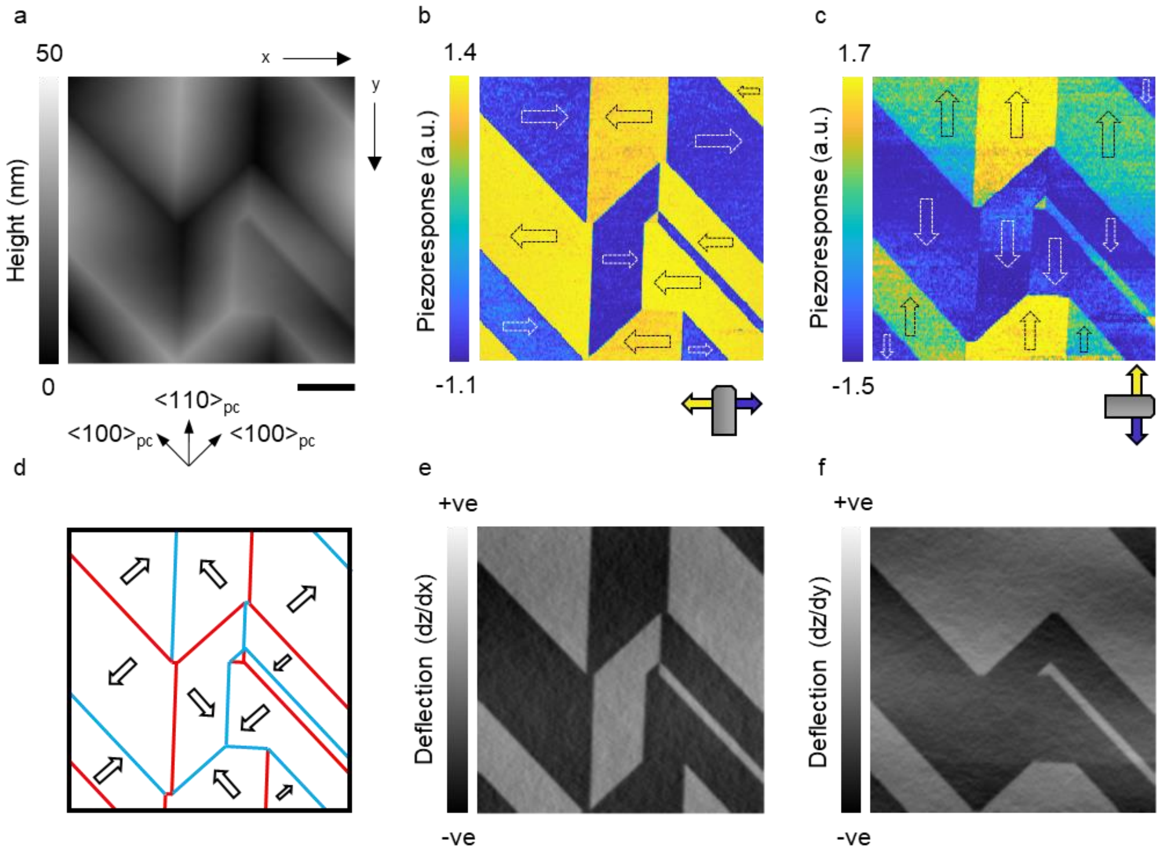

Supplementary Figure S3| Topography and lateral-PFM of Cu-Cl boracite. (a) Localised surface topography and (b,c) the corresponding as-measured lateral piezoresponse ($Asin\Theta$) maps, with the sample rotated 0° and 90° with respect to the cantilever long axis (denoted by the grey motif below each map). 5 V_{ac} at 20 kHz was used for domain imaging. Blue and yellow arrows indicate the link between the colours used in the $Asin\theta$ maps and the direction of in-plane tip movement and cantilever distortion. Black (and white) dotted arrows explicitly denote the conventionally inferred polarisation component. (d) Conventionally predicted orientation of the polarisation in the respective domains, using as-measured piezoresponse maps. Predicted head-to-head and tail-to-tail charged walls are indicated in blue and red, respectively. Such a polarisation map is inconsistent with symmetry considerations which demand that <100>$_{pc}$-oriented boundaries should be uncharged 180° walls (whereas here all of the 180° walls appear to be charged). (e,f) Topography derivative (rate of change of z-height with either the +x or +y directions). The sign of each derivative map can be reversed depending on whether the forward/reverse scan path is taken and maps unusually well to the lateral PFM phase information. The scale bar measures 5µm.

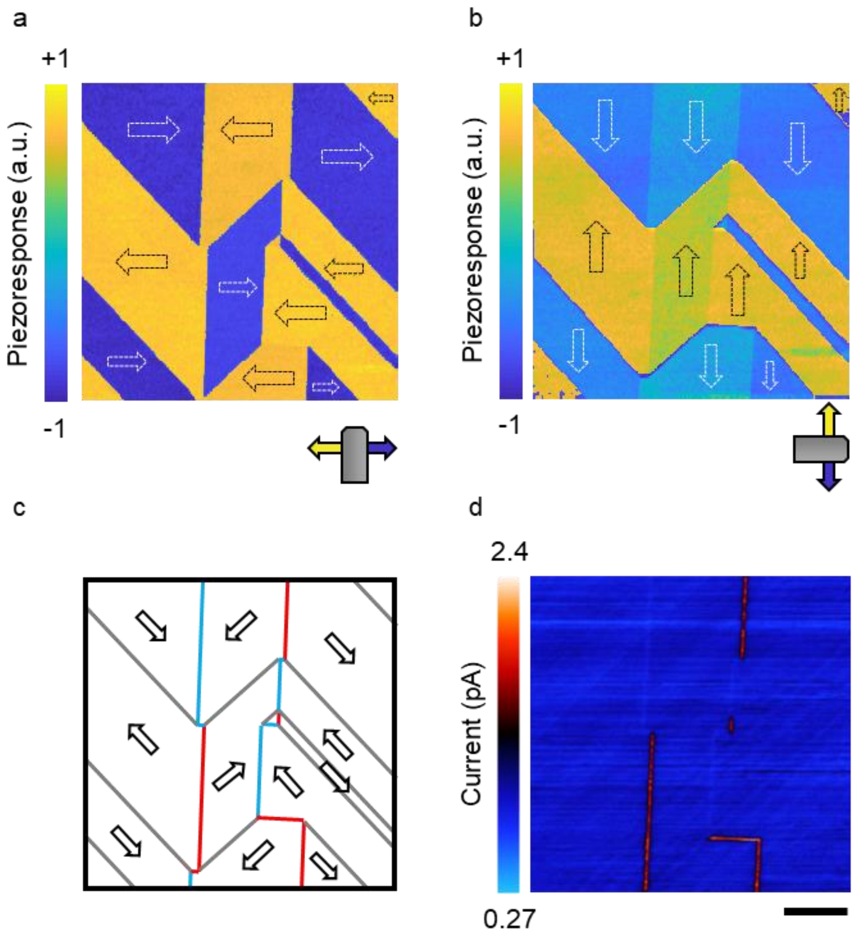

Supplementary Figure S4| Corrected lateral-PFM and as-measured cAFM. (a,b) Corrected piezoresponse ($Asin\Theta$) maps obtained by subtraction of a deflection-based cross-talk signal, established in previous work [McQuaid *et al.* Nat. Commun. **8**, 15105 (2017)]. Dotted-black and white lines explicitly denote the polarisation component determined for each domain. (c) Orientation of the polarisation in each domain after the cross-talk correction. These domain orientations are now consistent with both the known domain-wall types in the boracite system (uncharged 180° type and charged 90° type) and the charged wall profile inferred from the measured current map pattern (d). The faint bands that coincidentally align approximately along <100>$_{pc}$ in (d) are attributed to a mechanical polishing artefact as they do not spatially correlate with the underlying domain configuration. Scale bar measures 5 μm.

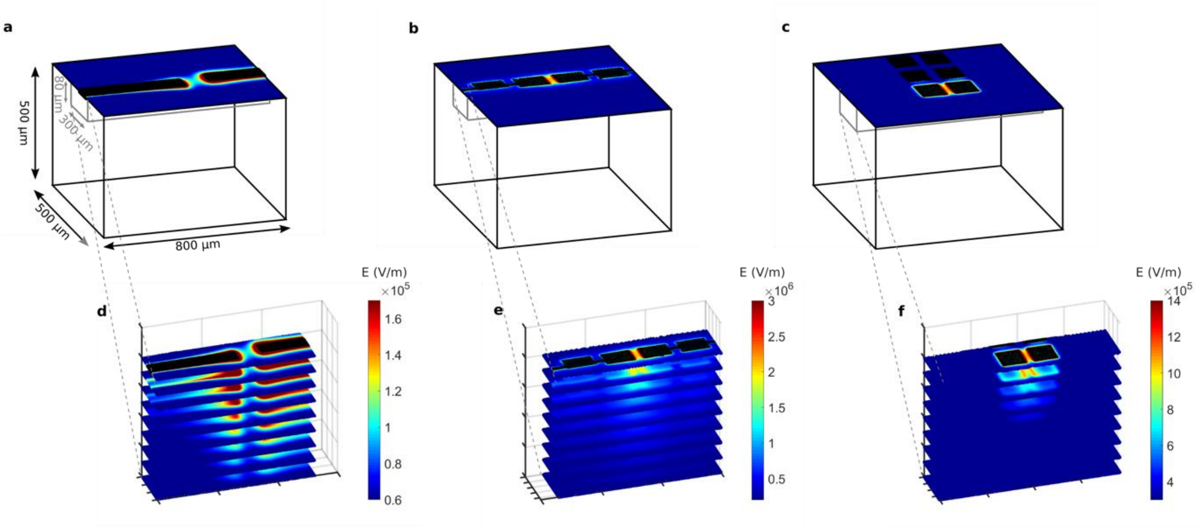

Supplementary Figure S5| Numerically simulated electric field distribution for various co-planar electrode geometries in which anomalous domain wall motion was observed. The main article presents observations of anomalous domain wall motion in Cu-Cl boracite, made using a simple coplanar electrode geometry (the black strips which can be seen on the specimen surface in the model representations in a and d above). In such simple cases, it is clear that the local change in the electrostatic energy, due to the wall motion (of the form E.P), must be positive. However, over the course of several years, the team has investigated anomalous wall motion using a number of different electrode geometries, positioned differently with respect to the boracite domains and domain walls. We have used numerical simulations (using COMSOL) to understand the form and extent of the electric fields generated for each electrode arrangement used, both at the top surface (a – c) and through the depth of the boracite crystal (d-f). Combining this information with the field-induced domain wall movements (monitored by in-situ optical microscopy) across the entire crystal, the change in the electrostatic energy associated with polar reorientation has been calculated at each point (numerical models generate the field at the point, while optical imaging determines the change in polarisation at the same point), and the global energy sum has been determined. In all cases, it is the energy change within the interelectrode gap that dominates and the overall calculated change in the electrostatic energy is always positive, when the field is applied to the head-to-head walls which move anomalously. This is true even in rather complex situations where electrodes have been placed near domain wall triple junctions, where fields cause both conventional motion of tail-to-tail 90° walls and anomalous motion of head-to-head walls at the same time (see supplementary movies M6-M7).

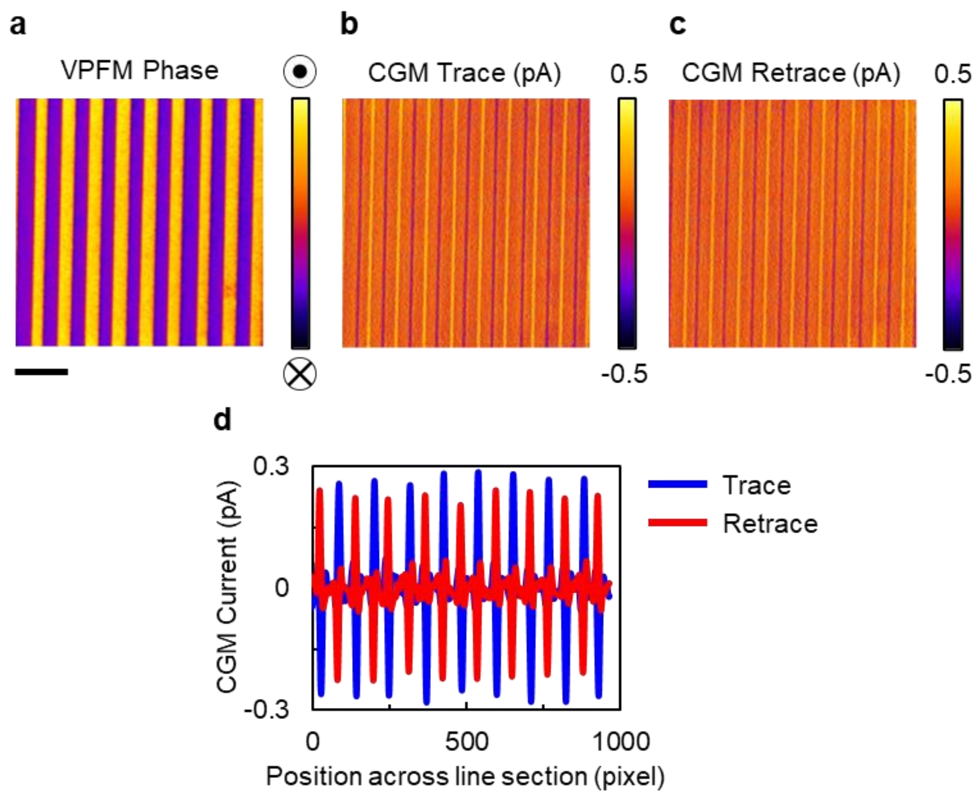

Supplementary Figure S6| CGM on periodically poled z-cut lithium niobate (PPLN). (a) Vertical piezoresponse force microscopy (VPFM) phase map (imaged with 1 V_{ac} near resonance) clearly illustrating the out-of-plane "up/down" nature of local 180° oriented domains. The scalebar measures 20 µm. (b) Corresponding CGM current trace (imaged with a scan speed of 9.77 Hz) reveals distinct current signals, located at domain walls and alternating in sign. (c) The current detected at each domain boundary is observed to flip in sign in the CGM current retrace image when compared to the trace image. These results agree qualitatively with those presented by Hong *et al.* PNAS **111**, 6566-6569 (2014). (d) Averaged current profiles for both trace (blue) and retrace (red) demonstrating the reversal in sign of current detected at each wall with reversal in tip-scan direction.

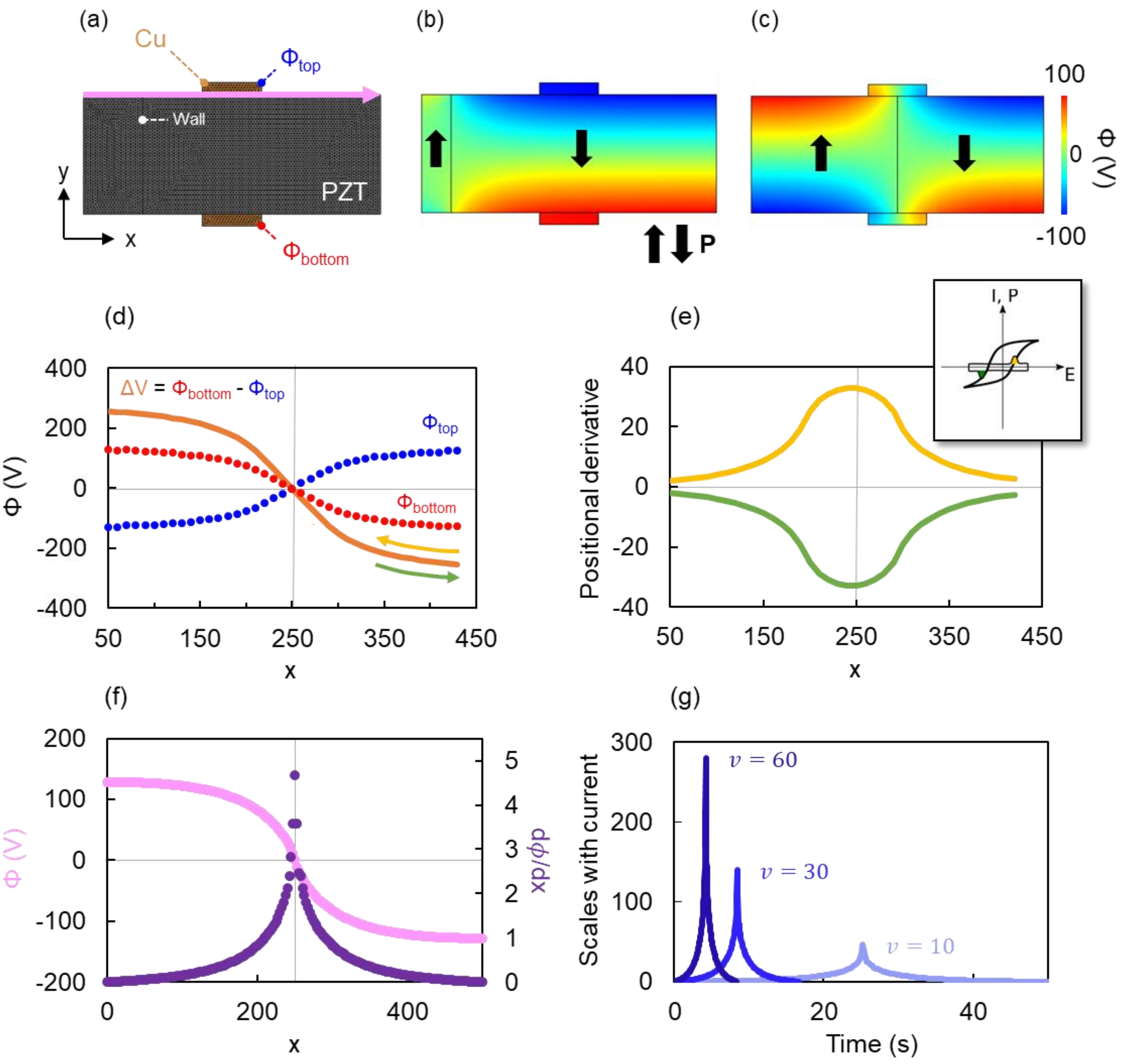

Supplementary Figure S7| Finite element simulations to reproduce CGM for out-of-plane polarised samples (a) Geometry of the simulations. The average of the electric potential on the top (Φ_{top}) and on the bottom (Φ_{bottom}) as a function of domain wall position is represented in (d). The electric potential on the top surface of the ferroelectric slab is presented in (f). (b,c) Two examples of the solved electrostatic model: electrostatic potential (Φ) maps for different positions of the wall. (d) Evolution of the electrostatic potential on the top (Φ_{top}, blue circles) and bottom (Φ_{bottom}, red circles) electrodes and the voltage build-up across the electrodes (ΔV, orange line). (e) Derivative of the voltage as a function of the position of the wall in the forward (yellow) and backward (green) directions. (f) Electrostatic potential (light pink) on the surface of the ferroelectric slab as a function of position for a wall at its centre and the positional derivative (violet), which scales with the current that would be measured in a CGM experiment. (g) Example of the effect of the tip velocity on the current peak – the peak height increases as velocity increases, but the integrated charge remains constant.

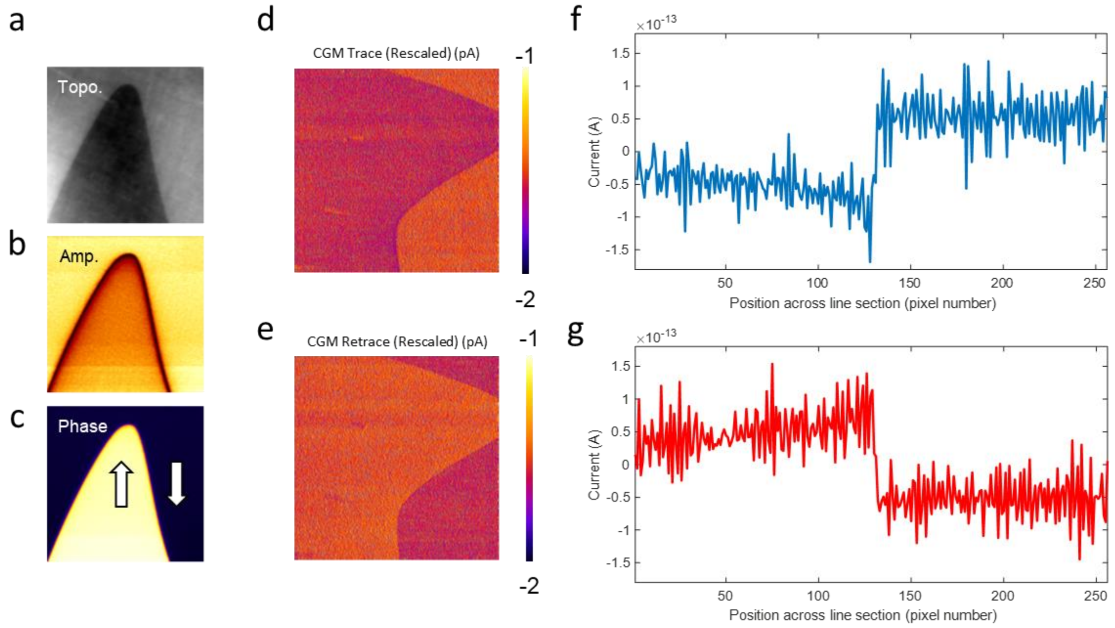

Supplementary Figure S8| In-plane CGM in y-cut LNO. (a-c) Lateral-PFM dataset comprising of topography, lateral amplitude, and phase respectively (used to establish the head-head nature of the walls in this particular cut). (d,e) In-plane CGM current trace and retrace, respectively, of the same general region mapped in a-c, illustrating domain current contrast unlike the domain wall current contrast observed in the out-of-plane orientation. An important feature is that the sense of current detected on the trace scan is reversed in the retrace scan. (f,g) Extracted CGM current line profiles from (d,e) reveal the step in current associated with traversing across one domain to the next and also that the current detected within each individual domain during CGM scanning is approximately constant.

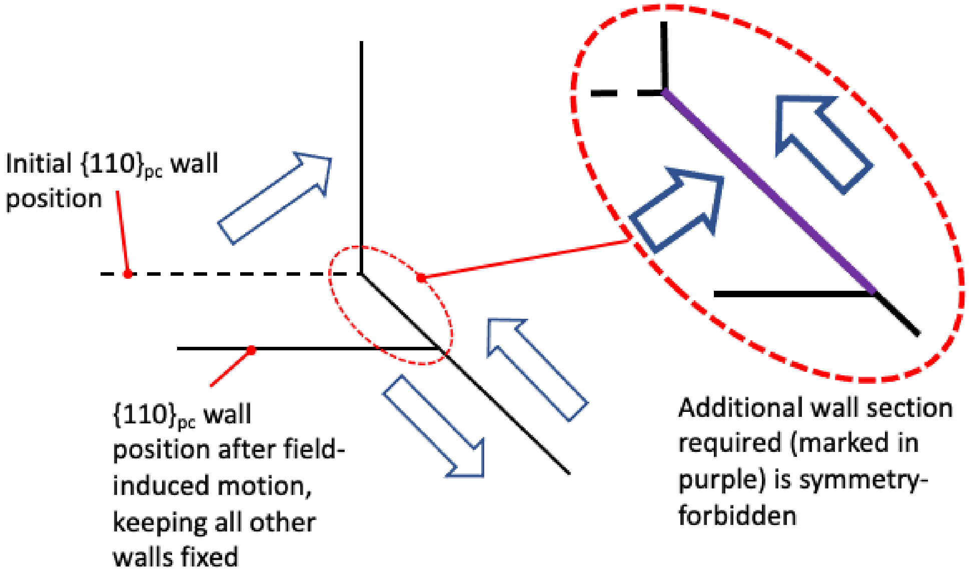

Supplementary Figure S9 | Multiple wall motion around domain wall junctions. Even though the electric field has only been applied to one of the domain walls in the three-fold domain wall junction region shown in Supplementary Movie 6, wall movement and changes in all three walls occur. This is needed in order to facilitate the field-driven motion of the wall to which bias is applied (the horizontal tail-to-tail wall in this instance). If other domain walls remain unchanged or unmoved, then symmetry-forbidden wall geometries necessarily result (seen in the area highlighted by the red-dotted ellipse and magnified in the inset). To avoid such symmetry-forbidden geometries, multiple wall motion is inevitable. In other words, when such junctions exist in the microstructure, field-driven wall motion necessarily leads to a coupled motion response from other walls in the microstructure. This is an unavoidable requirement of symmetry.

Supplementary Note S1: Interpretation of PFM data in boracites

Lateral piezoresponse force microscopy (PFM) offers an excellent means to identify in-plane polar variants for a crystal or thin film ferroelectric. For proper ferroelectrics, which generally exhibit large spontaneous polarisation (linked to large piezoelectric coefficients), the technique works in a straightforward manner for interpretation of polar variants. However, interpreting piezoresponse signals in ferroelectric systems with small polarisation, such as in some improper ferroelectrics, can pose a challenge due to weak piezoelectric responses. Confounding factors that depend on the material and/or instrumental setup can lead to erroneous results, due to e.g., off-axis coefficients contributing to lateral signal at large ac voltages, flexure dominating the actual signal and topographic crosstalk. In the case of PFM conducted on Cu-Cl boracite presented in this manuscript, we found that the PFM data needed to be corrected before domain orientations could be uniquely assigned. The methodology and the rationale for the correction have been discussed at length in the supplementary note 1 of the article McQuaid *et. al,* Nature Comm. **8**, 15105 (2017). Using the same methodology, the raw PFM data shown in figure S3 has been corrected to obtain the maps shown in figure S4.

Supplementary Note S2: Charge Gradient Microscopy

Charge gradient microscopy (CGM) is a relatively new scanning probe technique, first developed by Hong *et al.* [PNAS **111**, 6566-6569 (2014)], to map currents associated with spatial variations in ferroelectric polarisation, at the nanoscale. In the context of our work, we use CGM to infer and quantify the current that should be associated with the anomalous motion of head-to-head domain walls between fixed electrodes, under an applied bias. As the technique is new and not widely used and the mechanism for the generation of current observed in CGM is still under debate, we first validate our experimental procedure and numerical model on out-of-plane polarised periodically poled lithium niobate (PPLN), before moving on to explore and understand the contrast mechanism in the more complex in-plane polarised lithium niobate (LNO) and improper ferroelectric $Cu_3B_7O_{13}Cl$ (copper-chlorine boracite) cases.

S2.1 Out-of-plane polarised sample (z-cut periodically poled $LiNbO_3$)

S2.1.1 Experimental methods and results

CGM involves quickly rastering a grounded, conducting AFM probe across a grounded sample with a large deflection setpoint (*i.e.* tip pressure) while passively measuring any current that flows. This method of imaging was first reported on periodically poled z-cut lithium niobate (PPLN), where the large spontaneous polarisation (P_S≈80 µCcm^{-2}) is perpendicular to the surface and points alternately upwards and downwards. In their original work, Hong *et al.* observed a strong current at the domain walls, the sign of which depended on the direction of rastering. Integrating this current with respect to time is, by definition, the charge variation observed at the tip (ΔQ). This charge variation was suspected to reflect the change in the spontaneous polarisation (P_S) either side of the domain wall, and this was quantitatively demonstrated in the original work. In this context, CGM can be seen as a way to measure the switching currents associated with the reversal of polarisation in a ferroelectric, without the application of an electric field or the need for any domain wall movement in the laboratory frame of reference. We performed CGM by scanning a grounded platinum (Pt) tip (RMN 25Pt200B) over the surface of commercially available PPLN, which was also grounded. Scans were performed with a total deflection setpoint of 18V. The results are presented in supplementary figure S6. Current peaks associated with the electrode passing over domain walls can be clearly observed. Our findings therefore clearly mirror those of the original study by Hong *et al.*.

The origin of the currents observed in CGM are as yet unclear. It seems that two possibilities exist:

(i) the high-tip pressure scanning scrapes any atmospherically generated screening charges away from the ferroelectric surface, revealing pristine uncompensated material, or that it simply pushes through any surface film of compensating molecules, such that clean and direct contact is made with the ferroelectric surface. Either way, charges compensating the bound charge of the ferroelectric then develop on the metallic CGM tip and change in their distribution and sign as the tip passes over differently oriented domains;

(ii) the scanning tip has complex interactions with the screening charges themselves, rather than interacting directly with the ferroelectric surface.

The need for several scans to be conducted before good CGM signals are obtained and the dramatic changes in the surface potential before and after CGM [Tong *et al.* ACS Nano 10, 2568-2574 (2016)] hint at a need to *remove* surface adsorbate to generate CGM signal and therefore suggest that a direct charge compensation in the tip may be a more robust explanation for the observed currents (as mooted by Gregg and Kumar, Nature, **510**, 481 (2014)). Irrespective of these details, however, the team that invented CGM have clearly shown that the contrast can be used to monitor the switching currents that would be seen if the domain walls were to move relative to a stationary electrode as a result of bias-induced switching. To reinforce this notion, we decided to compare measured CGM currents to expectations from classical electrostatics, by conducting finite element modelling using COMSOL Multiphysics software.

S2.1.2 Numerical methods and results

The two-dimensional model, illustrated in figure S7, represents a ferroelectric with dimensions 500 x 200 μm^2, with two small electrodes on each side of the short axis. The material under test was chosen to be lead zirconate titanate (PZT – a choice for which physical properties are included in the software database), with a dielectric permittivity of ~1,700. Both electrodes were assigned to be copper, with a conductivity of $6x10^7$ Sm^{-1}. By default, COMSOL does not account for spontaneous polarisation; the associated physics was added separately as a remnant displacement D_r such that the total displacement D is given by $D = \varepsilon_o \varepsilon_r E + D_r$. The remnant displacement was oriented along the vertical (y-axis) and set to 20 μCcm^{-2}. For the two ferroelectric domains outlined above, the direction of the remnant displacement was manually reversed, and the position of the boundary was also varied continuously across the ferroelectric slab. The mesh was created using the automated “physics-controlled” tool and chosen to be “extremely fine”. The largest dimension of all triangle mesh elements was smaller than 5 μm.

According to classical electrostatics, the normal component of the electrical displacement across the boundary between two insulators is continuous, provided that there is no charge accumulation at the boundary [see for example von Hippel's textbook "Dielectric Materials and their Applications"]. In a perfect metal, an electric displacement cannot be maintained and is nominally zero. Were the metal to form a boundary with a dielectric, a strong discontinuity at the interface would emerge. In our model, we impose the continuity of the electric displacement at the dielectric-metal interface. This is plausible in the case where the electrodes are connected to an infinite source or sink of charges *e.g.* connected to the Earth as it is the case in the CGM experiment.

Using this geometry, the model was then solved for the stationary (*i.e.* electrostatic) case. Several models were constructed for the wall being sequentially repositioned from one side to the other (to reproduce the variations in the separation between domain wall and electrode that would be mapped during a CGM scan). Two examples are presented in supplementary figure S7 (b and c). Since the solution is obtained in the static time-independent case, no information about current flow can be directly obtained. However, the electrostatic potential, ϕ, can be computed everywhere and the potential difference (voltage) $V = \Delta\phi$ between electrodes can be calculated a posteriori. The current signature between the electrodes, induced by the wall moving at constant speed, can then be inferred from the change in voltage between any two wall positions, as will be discussed later.

As a first step, we can take a look at the case when the wall is far from the electrodes (figure S7 (b)); this is similar to the situation when there is a single domain only. The remnant displacement in the ferroelectrics induces charge separation across the ferroelectric slab leading to different electric potentials at each electrode and therefore the build-up of a voltage. Turning to the case when the wall is directly under the electrodes (supplementary figure S7(c)), it can be clearly observed that one half of the electrode feels one direction of polarisation (assigned as 'up'), while the other half reacts to the opposite direction of polarisation ('down'). This leads to a variation of the electric potential across each electrode. Since the wall is perfectly at the centre in this case, the electric potential in the electrode created by one domain will be equal and opposite in sign to the one created by the opposite domain, leading to a zero average electrostatic potential within each electrode (supplementary figure S7(d)), and consequently to an overall zero voltage drop between the two electrodes.

The voltage developed between the electrodes (orange line in supplementary figure S7(d)) continuously varies with the position of the wall, from the value for a fully polarised ferroelectric slab in one direction to zero when the wall is at the centre all the way to the value found for the fully polarised case with polarisation in the opposite direction, with a slight change in curvature when the

wall is on the edge of the electrodes. Therefore, finite element modelling directly informs us of the evolution of voltage across the ferroelectric slab, as a function of the wall position. To obtain knowledge regarding the current associated with the domain wall motion, it is, however, necessary to do further analysis. Indeed, since the simulations are run in the open-circuit steady-state regime, no charge flow is directly modelled. However, we know that, in a short-circuited situation, similar to the CGM experiment, charges will flow when the wall is moving to compensate for the change in voltage at the electrodes. The amount of charge needed in response to the change in voltage is given as $\Delta Q = C\Delta V$. Hence, the current I that will be associated with the domain wall motion can be derived as:

$$I = \frac{\Delta Q}{\Delta t} = C\frac{\Delta V}{\Delta t} = C\frac{\Delta V}{\Delta x}\frac{\Delta x}{\Delta t} = C\frac{\Delta V}{\Delta x}v \qquad \text{eq (S1)}$$

where v is the wall velocity. In other words, the current associated with the wall motion is directly proportional to the derivative of the voltage across the ferroelectric slab with respect to the wall position (supplementary figure S7(e)). It is worth noting that the current reverses depending on the direction along which the derivative is taken, which makes intuitive sense for ferroelectric switching. Indeed, textbook ferroelectric P-E loops (inset) show that the current associated with the switching from up-to-down polarisation is opposite to the down-to-up polarisation switching.

The approach taken so far models the electrostatic response for a ferroelectric sandwiched between two electrodes, which is a fairly reasonable depiction of the CGM experiment where the tip acts as a pick-up electrode and the bottom of the sample is grounded. We can push our model further to describe a perfect point contact, by considering the electrostatic potential on the top surface when the wall is at the centre of the slab (supplementary figure S7(f)). Applying the same approach to estimate the current that would be produce in the CGM experiment, the derivative of the electrostatic potential as a function of the position on the surface can be taken (supplementary figure S7(f)). Unsurprisingly, the current peaks more sharply than in the case of the extended electrodes, as the "sensing" electrode is now a point contact. However, the overall form of the response remains the same. A real experiment will have a tip that is broader than a point contact but not quite as extended as in the model with the fixed electrodes; the current peak will therefore be slightly more rounded than modelled with a single point and much sharper than calculated for microns-wide electrodes.

It is worth noting that our electrostatics model cannot reproduce the effect of velocity. However, it is clear from equation (S1) that the current measured will scale with the velocity v. This is exemplified in supplementary figure S7(g) where the CGM peak current increases with increasing velocity: the faster the wall moves, the higher the current peaks when the wall goes under the

electrodes, following eq. (S1). The peak, however, extends over longer time scale when the velocity is decreased, to maintain the area under the curve (switched charge) constant, *i.e.* regardless of the velocity, the total charge picked up by the electrodes is constant. This reproduces the observation of linear dependency of measured current and tip velocity reported by Hong *et al.* and also seen in our work.

In summary, this finite element model, accounting for classical electrostatics, can be used to readily explain the CGM current observed in out-of-plane polarised z-cut PPLN. Indeed, the positional derivative of the electrostatic potential at the surface of a ferroelectric slab is directly proportional to the CGM current.

S2.1.3 From current to polarisation

Now that we understand the generation of the CGM current, the analysis of the experimental results can be pushed further by calculating the associated change of polarisation ΔP.

$$\Delta P = \frac{\Delta Q}{A} \text{ where } \Delta Q = \int I \cdot dt = \sum I \cdot \Delta t$$

With ΔQ the charge variation, I the CGM current, t time and A is the area of the tip that can be estimated from the radius of the tip given by the provider. The dwell time at each pixel Δt can be directly calculated from the experimental parameters following $\Delta t = 1/(2n \cdot f)$,where f is the frequency of the scan and n is the number of pixels per fast axis. The sample-probe contact area A is given by πR^2 where R is the probe radius. For a probe radius R of ~20nm, this only gives 4 μCcm^{-2} in our case. This value is significantly smaller than the spontaneous polarization reported for bulk $LiNbO_3$. Qualitatively, our results agree with those presented by Hong *et al.*; the discrepancy in the quantitative polarisation calculation (Hong *et al.* get values close to that of bulk $LiNbO_3$) may be because our adaptation of the AFM for current pick-up in CGM may not be fully optimised.

As an intermediate summary, we showed that we can reliably measure the form of the current associated with out-of-plane polarisation reversal using CGM (even if our absolute currents are lower than expected and lower than those seen in the equivalent scans performed by Hong *et al.*). We have interpreted CGM results in terms of classical electrostatics and this approach seems to explain both our results and the results reported in the literature to date. Hence, we have established that CGM can be used to observe the change of out-of-plane polarisation at a domain wall. This can be seen as conducting a spatial polarisation loop where the reversal of the polarisation is not induced by the application of an electric field but by changing the position of the tip electrode.

S2.2 In-plane polarised LNO with head-to-head polar inversion

We can move to the more challenging case of using CGM as used to measure switching current induced by in-plane domain reversal. With in-plane polarisation, there are no conventional bound charges at the surface to create an obvious electrostatic potential that needs to be screened. However, measurements conducted on in-plane polarised head-to-head walls in LNO show constant low-level currents within domains and a distinct current step at the boundary between the two domains (supplementary figures S7). This can be explained readily by revisiting classical electrostatics.

The finite element model developed above was adapted to the case where polarisation is in-plane. The geometry and boundary conditions are kept the same and the only major difference is the orientation of the domain wall: which is now parallel to the electrodes. The two extreme cases are similar to those discussed in the out-of-plane case: if the ferroelectric is fully polarised, the voltage build up between the electrodes is that of the single domain case; when the wall is exactly at the centre, the two domains are of the same size and there is no voltage dropped between the electrodes. However, importantly in this case the two electrodes are not at a null electric potential and this leads to a very different evolution of the voltage with the position of the wall.

When the position of the wall is moved away from the centre, the relative thickness of the two domains is continuously changing. As the voltage developed across a single domain varies linearly with the thickness of the domain, the change of voltage across the electrodes also varies linearly with the position of the wall. This can be easily understood considering the change of polarisation of the entire ferroelectric slab: when the wall moves, the polarisation changes by $2P_s \cdot \Delta y$. This linear dependence of the voltage with the position can be clearly seen in figure 3 of the main manuscript.

Now taking the same approach as before, the flow of charge associated with scanning a CGM tip across the surface in-plane head-to-head wall can be calculated by taking the positional derivative of the electrostatic potential. Figure 3 in the main manuscript presents the electrostatic potential and its derivative as a function of the position for a wall. The linear dependence of the electrostatic potential can be well seen on each side of the wall with the sign of the slope related to the direction of polarisation. It follows that the derivative on each side of the wall is constant with a sign change between the domains. In other words, the charges that are necessary to compensate for the change in electrostatic potential when the electrode scans across the surface would have been constant in each of the domains and change sign at the wall.

The magnitude of the associated current can be written as

$$I = \frac{\Delta Q}{\Delta t} = C\frac{\Delta V}{\Delta t} = C\frac{\Delta V}{\Delta \mathrm{y}}\frac{\Delta \mathrm{y}}{\Delta t} = C\frac{\Delta V}{\Delta y}v = Cv\frac{2P_S \cdot \Delta y}{\Delta \mathrm{y}} = 2Cv\,P_s \quad \text{eq (S2)}$$

Once again, we observed the linear dependency between the current and the tip velocity. Additionally, the linear dependence of the current with spontaneous polarisation in the case of in-plane polarised CGM current is apparent. Therefore, we showed that classical electrostatics predicts that in a CGM experiment on a head-to-head wall the current measured will be constant in each of the domains and a step associated with the change of sign will be observed across the wall. The magnitude of the current in each of the domains will scale with both tip velocity and the sign and magnitude of the spontaneous polarisation. These features are fully reflected in the experiment (figure S7).

Captions for the Supplementary Movies

Supplementary Movie 1
Transmission mode optical microscopy (in polarised light) of a 90° domain wall in Cu-Cl boracite. The video shows how the contrast from the wall changes as the focus is varied from the top to the bottom surface of the crystal. As its position relative to the electrodes deposited on the top surface does not change, it appears that the plane of the wall is genuinely orthogonal to the surface. A series of still images are presented in figure S2 illustrating the same thing.

Supplementary Movie 2
Transmission mode optical microscopy (in polarised light) of a tail-to-tail 90° charged conducting wall moving under the application of a long-period triangular voltage waveform, at a temperature of 84 °C. As can be seen, the wall moves in the opposite sense to that of the applied field, as should be expected.

Supplementary Movie 3
Transmission mode optical microscopy (in polarised light) of a head-to-head 90° charged insulating wall, moving under the application of a long-period triangular voltage waveform, at a temperature of 84 °C. This wall also moves in the opposite sense to that of the applied field. In this case, the motion is described as anomalous, as it is associated with the growth of the domain with components anti-aligned with the field and the associated contraction of that with aligned polar components.

Supplementary Movie 4
Transmission mode optical microscopy (in polarised light) of both tail-to-tail and head-to-head 90° charged walls. As can be seen, and as should be expected from M2 and M3, they both move in the same direction under application of a long-period triangular voltage waveform (again at a temperature of 84 °C). This is conclusive evidence that anomalous domain wall motion is occurring.

Supplementary Movie 5
Transmission mode optical microscopy showing a head-to-head 90° charged domain wall moving under application of a DC bias at a temperature of 84 °C. Reversible reorganisation of the domain microstructure can be seen some distance away from the interelectrode gap, where electric fields are expected to me minimal.

Supplementary Movie 6
Transmission mode optical microscopy (in polarised light) of a tail-to-tail 90° charged wall moving under application of an alternating positive/negative voltage of fixed magnitude at a temperature of 84 °C. Clearly, both the tail-to-tail and the perpendicular head-to-head walls move as a result, as does the three-fold junction with an uncharged 180° domain wall. This coupled movement is rationalised, to some extent, in figure S9. The video playback rate has been increased by a factor of 10.

Supplementary Movie 7
Transmission mode optical microscopy (in polarised light) of a head-to-head 90° charged wall moving under application of an alternating positive/negative voltage of fixed magnitude at a temperature of 90 °C. Again, field-induced movement is associated with movement of the perpendicular tail-to-tail 90° wall and the triple junction with the uncharged 180° wall. The video playback rate has been increased by a factor of 10.
